# Impaired Trunk Stability in Individuals at High Risk for Parkinson's Disease

**DOI:** 10.1371/journal.pone.0032240

**Published:** 2012-03-23

**Authors:** Walter Maetzler, Martina Mancini, Inga Liepelt-Scarfone, Katharina Müller, Clemens Becker, Rob C. van Lummel, Erik Ainsworth, Markus Hobert, Johannes Streffer, Daniela Berg, Lorenzo Chiari

**Affiliations:** 1 Center of Neurology, Hertie Institute for Clinical Brain Research, Department for Neurodegenerative Diseases, University of Tübingen, Tübingen, Germany; 2 DZNE, German Center for Neurodegenerative Diseases, Tübingen, Germany; 3 Department of Geriatric Rehabilitation, Robert-Bosch-Hospital, Stuttgart, Germany; 4 Department of Electronics, Computer Science and Systems, University of Bologna, Bologna, Italy; 5 McRoberts, The Hague, The Netherlands; 6 Janssen Research and Development, Janssen – Pharmaceutical Companies of Johnson & Johnson, Beerse, Belgium; Philadelphia VA Medical Center, United States of America

## Abstract

**Background:**

The search for disease-modifying treatments for Parkinson's disease advances, however necessary markers for early detection of the disease are still lacking. There is compelling evidence that changes of postural stability occur at very early clinical stages of Parkinson's disease, making it tempting to speculate that changes in sway performance may even occur at a prodromal stage, and may have the potential to serve as a prodromal marker for the disease.

**Methodology/Principal Findings:**

Balance performance was tested in 20 individuals with an increased risk of Parkinson's disease, 12 Parkinson's disease patients and 14 controls using a cross-sectional approach. All individuals were 50 years or older. Investigated groups were similar with respect to age, gender, and height. An accelerometer at the centre of mass at the lower spine quantified sway during quiet semitandem stance with eyes open and closed, as well as with and without foam. With increasing task difficulty, individuals with an increased risk of Parkinson's disease showed an increased variability of trunk acceleration and a decrease of smoothness of sway, compared to both other groups. These differences reached significance in the most challenging condition, i.e. the eyes closed with foam condition.

**Conclusions/Significance:**

Individuals with an increased risk of Parkinson's disease have subtle signs of a balance deficit under most challenging conditions. This preliminary finding should motivate further studies on sway performance in individuals with an increased risk of Parkinson's disease, to evaluate the potential of this symptom to serve as a biological marker for prodromal Parkinson's disease.

## Introduction

For the progressive neurodegenerative disorder Parkinson's disease (PD), neuromodulatory or even neuroprotective therapy could soon be available. The best effect of such a therapy will undoubtedly be achieved when administered in the earliest as possible disease phase. In order to accurately test neuroprotective effects, potential drugs need to be challenged with markers of disease progression. These markers, however, are not yet available to a sufficient extent and quality [1].

Clinical PD is a disease with motor dysfunction as the leading symptom. Postural instability is, as one of the four cardinal motor features, part of this motor dysfunction. Until recently, it has been considered to occur relatively late in the disease course. This is reflected by the Hoehn&Yahr scale where “postural instability” is represented only in the advanced stages 3 to 5 [2]. However, there is accumulating evidence that changes of postural stability occur even at early PD stages [3], [4], [5], and that postural instability increases when PD deteriorates [6].

From a clinical point of view, there is no doubt about the existence of prodromal motor symptoms. This is what the clinicians experience from newly diagnosed PD patients who report, e.g. about a history of reduced arm swing and reduced ability to turn in difficult situations. In addition, people with highly trained motor skills such as musicians and top athletes who do not yet have PD, occasionally report about slowly progressive problems in performing their movements in the usual velocity and accuracy. As an example of such early changes, reduced movement of Ray Kennedy's right arm was observable in videos of soccer games up to eight years before PD was diagnosed [7]. A recent study found altered gait parameters in LRRK2 G2019S mutation carriers without a clinical diagnosis of PD [8]. This mutation leads to a Parkinsonian syndrome with relatively high probability. Based on their mean age at study inclusion (53 years) and the knowledge about the penetrance of the LRRK2 gene (28% at age 59 years [9]), approximately one out of four of their study participants with a LRRK2 mutation will develop clinical PD within the following six years. The finding that gait parameters in individuals at increased risk for PD are altered in combination with the probable association of gait and sway changes in PD [10] make it intriguing to hypothesize that also sway parameters may be changed in individuals at increased risk for PD.

Besides the occurrence of motor deficits the prodromal phase of PD is associated with an increased probability of the occurrence of non-motor symptoms such as depression, hyposmia, REM sleep behaviour disorder (RBD), and of signs such as an enlarged hyperechogenicity of the substantia nigra already indicating neurodegenerative decline [11], [12], [13]. In more detail, depression is associated with a 3-fold increased risk for future PD [14], hyposmia with a 5-fold increased risk [15], and an enlarged hyperechogenicity of the substantia nigra with an approximately 18-fold increased risk for future PD [16]. There is increasing evidence that the combined occurrence of these factors could even increase the risk for PD and that these individuals represent a high risk group for PD (HR-PD) [17], [18], [19], [20], [21].

In this study we investigated sway of such HR-PD individuals to test the hypothesis that the postural control system is affected already at a prodromal stage of PD. As compensatory mechanisms can make subtle changes of primary damaged networks invisible [22], [23] we used a demanding paradigm, and included parameters of postural correction in the analysis.

## Methods

### Ethics

The study protocol was approved by the ethical committee of the Medical Faculty of the University of Tuebingen, and all individuals provided written informed consent.

### Objective

The primary objective of the study was to test whether trunk instability as a sign for subtle balance deficits even occur at prodromal stages of PD. Secondary aim was to exploratively analyze associations of trunk balance parameters with demographic and clinical parameters of the subgroups.

### Individuals

A population of 20 individuals at high risk for future PD (HR-PD individuals), 12 PD patients (all OFF-medication), and 14 controls were investigated in the frame of the PMPP study (*Progression markers in the suspected prediagnostic phase of Parkinson's disease*). PD patients were diagnosed according to established diagnostic criteria [24], and were only included if they had a Hoehn&Yahr stage ≤2.5 (i.e. no clinical signs of postural instability), were older than 50 years of age, had no deep brain stimulation, and neither a history nor actual signs of a psychiatric disease. Controls fulfilled the following criteria: they were more than 50 years old, had a negative family history for PD [25] and no signs for PD [24], a normal area of hyperechogenicity of the substantia nigra [18], no history or actual signs of a psychiatric disease, and no signs of hyposmia [26].

PD diagnosis was also excluded in HR-PD individuals [24]. In addition, HR-PD individuals were defined as having the following symptom/factor constellations: (1) the presence of an enlarged area of hyperechogenicity of the substantia nigra (SN+ [18]) (all), and the additional occurrence of (2a) one PD cardinal motor sign - bradykinesia (N = 12) or rigidity (N = 7) as assessed with the Unified Parkinson's Disease Rating Scale (UPDRS) motor part [17], irrespective of the co-occurrence of signs/risk factors as mentioned in (2b) -, or (2b) two of the following signs/risk factors: lifetime depression (N = 7, according to the DSM-IV criteria) , hyposmia (N = 6) [26], reduced arm swing (N = 8), and positive family history of PD (N = 12) [25]. RBD was not used as a particular inclusion/exclusion criterion. In the RBD questionnaire, three PD patients, one HR-PD individuals, and one control scored >5 points which is suggestive of RBD.

Prior to sway measurement, all individuals underwent thorough examination by neurologists experienced in the field of neurodegenerative diseases, semiquantitative motor evaluation (UPDRS motor part), cognitive testing (Mini-Mental State Examination, MMSE), evaluation of depressive symptoms (Beck's Depression Inventory, BDI), and quantification of pallaesthesia at the malleoli using a 128-Hz tuning fork (WM, DB). None of the individuals had a medical history of, or suffered from clinically detectable polyneuropathy. For details see table 1.

**Table 1:** Demographic, clinical and sway parameters.

	PD	Individuals at high risk for PD	Controls	P-value
Individuals (females)	12 (5)	20 (7)	14 (7)	0.71
Age [ys]	61.5 (2.2)	61.9 (1.5)	63.9 (1.9)	0.53
Weight [kg]	79.3 (3.0)	78.2 (2.3)	72.6 (2.7)	0.18
Height [m]	1.74 (0.02)	1.73 (0.02)	1.72 (0.02)	0.80
BMI [kg/m^2^]	26.2 (0.8)	25.9 (0.6)	24.6 (0.7)	0.24
Age at disease onset [ys]	57.9 (2.1)			
Disease duration [ys]	4.3 (2.6)			
Hoehn&Yahr [1–5]	2.0 (0.4)			
UPDRS III [0–100]	26.5 (10.9)	3.3 (2.4)#	1.1 (1.7)#	<0.0001
MMSE [0–30]	29.2 (1.0)	29.2 (1.1)	29.7 (0.5)	0.23
SN hyperechogenicity (cm^2^)	0.24 (0.03)	0.24 (0.03)	0.14 (0.05)# †	<0.0001
BDI [0–63]	10.2 (8.5)	5.9 (5.9)	3.5 (3.7)#	0.02
Pallaesthesia [0–8]	7.3 (1.8)	7.1 (1.4)	7.9 (0.3)	0.17
ECF condition RMS ap (m/s^2^)	0.19 (0.12)	0.35 (0.17)#	0.17 (0.05)†	0.003
ECF condition JERK ap (m^2^/s^5^)	0.05 (0.04)	0.30 (0.37)#	0.05 (0.04)†	0.01
ECF condition RMS ml (m/s^2^)	0.18 (0.08)	0.28 (0.15)	0.17 (0.05)	0.03
ECF condition JERK ml (m^2^/s^5^)	0.13 (0.11)	0.56 (0.62)#	0.09 (0.10)†	0.01

Data are presented with the mean and standard deviation. P-values were assessed by ANOVA with post-hoc Student's t test, or with the Pearson test.

# p<0.017 compared to Parkinson's disease (PD),

† p<0.017 compared to individuals at high risk for PD.

ap, anteroposterior; BDI, Beck's Depression Inventory; BMI, Body Mass Index; ECF, eyes closed with foam; ml, mediolateral; MMSE, Mini Mental State Examination; RMS, root mean square of sway; SN hyperechogenicity, mean area of hyperechogenicity of the substantia nigra; UPDRS III, motor part of the Unified Parkinson's disease Rating Scale.

### Sway protocol

Participants were asked to stand upright in closed semitandem stance, feet were allowed to be externally rotated for comfortable standing, and arms flagged in self-chosen comfortable position. Prior to sway measurements, participants were asked to perform the most difficult task – eyes closed with foam (ECF) – to make sure that under experimental conditions the tasks could be adequately performed. All control and HR-PD individuals and all but two PD patients were able to perform it. These two PD patients were excluded from further analysis. Sway was assessed with an inertial sensor with 100 Hz sample frequency (DynaPort Hybrid, McRoberts, The Hague, the Netherlands), fixed with an elastic belt at the level of the third and fourth lumbar spine segment close to the centre of mass [27]. The sensing axes were oriented along the anatomical anteroposterior (AP), mediolateral (ML) and vertical directions. Four 30 seconds trials were performed: i) eyes open condition (EO) with gaze straight ahead at a white wall 2 meters in front, ii) eyes closed (EC), iii) eyes open with foam (EOF; Airex balance pad, 50×41×6 cm), and iv) ECF. The order of these conditions was randomly assigned for each participant to omit a systemic bias due to learning effects. Investigators were not specifically informed about the health status of HR-PD and control individuals.

### Data analysis and statistics

Pre-processing of acceleration signals has been previously described in [5]. In summary, acceleration signals were transformed to a horizontal-vertical coordinate system [28] and filtered with a 3.5 Hz cut-off, zero-phase, low-pass Butterworth filter. Then, the following parameters were evaluated from the acceleration signals measured with the inertial sensor in the AP and ML direction: Root mean square (RMS) of sway acceleration, mean sway velocity (MV), frequency comprising 95% of the signal (F95), and sway jerkiness (JERK) [5].

Statistical analysis was done with JMP 9.0, SAS. Data are presented with mean and standard deviation. P-values were assessed with ANOVA with post-hoc Student's t test (continuous data) or the Pearson Chi Square test, and were considered significant at a 0.05 level. Associations of the two most relevant outcome parameters of this study, i.e. RMS and JERK in ECF condition, with demographic/clinical parameters (independent variables: cohorts, age, gender, weight, length, UPDRS motor part, MMSE, BDI, SN status and pallaesthesia) were tested by use of a linear and logistic regression model.

## Results

HR-PD individuals were comparable to controls and PD patients regarding age, gender, weight, height, MMSE score and pallaesthesia. They did not differ significantly from controls regarding UPDRS motor score and BDI score. As expected, both HR-PD and control individuals had lower UPDRS motor scores than PD patients. PD patients had a higher (worse) BDI score than controls (table 1).

In the sway paradigm, with increasing task difficulty, HR-PD individuals showed an increase of RMS values in both the AP and the ML direction, compared to both control and PD individuals. This difference reached significance in the most challenging condition. Controls and PD patients did not differ significantly in either task.

**Figure 1:**
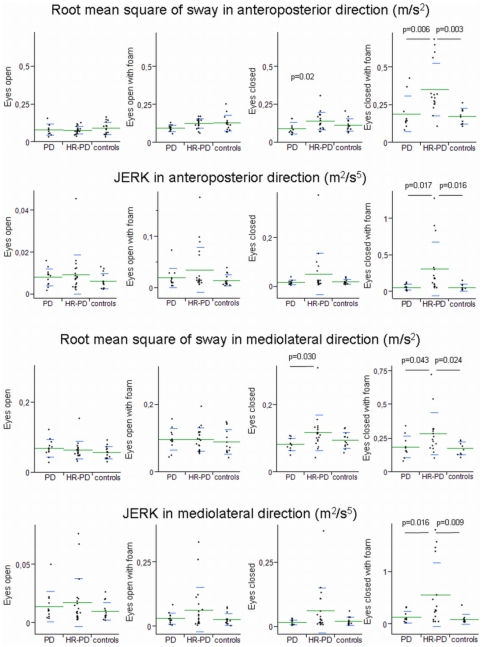
Root mean square acceleration and JERK results. Root mean square acceleration and JERK in the anteroposterior direction, and mediolateral direction, of 12 patients with Parkinson's disease (PD), 20 individuals at increased risk for PD (HR-PD), and 14 controls when performing increasingly difficult sway tasks. Note the different scaling in some of the graphs.

HR-PD individuals showed an increase of JERK values with increasing difficulty of the sway task which also reached significance in the most challenging condition, i.e. the ECF condition (table 1, figure 1).

MV and F95% were comparable between groups under all conditions tested (not shown).

**Figure 2:**
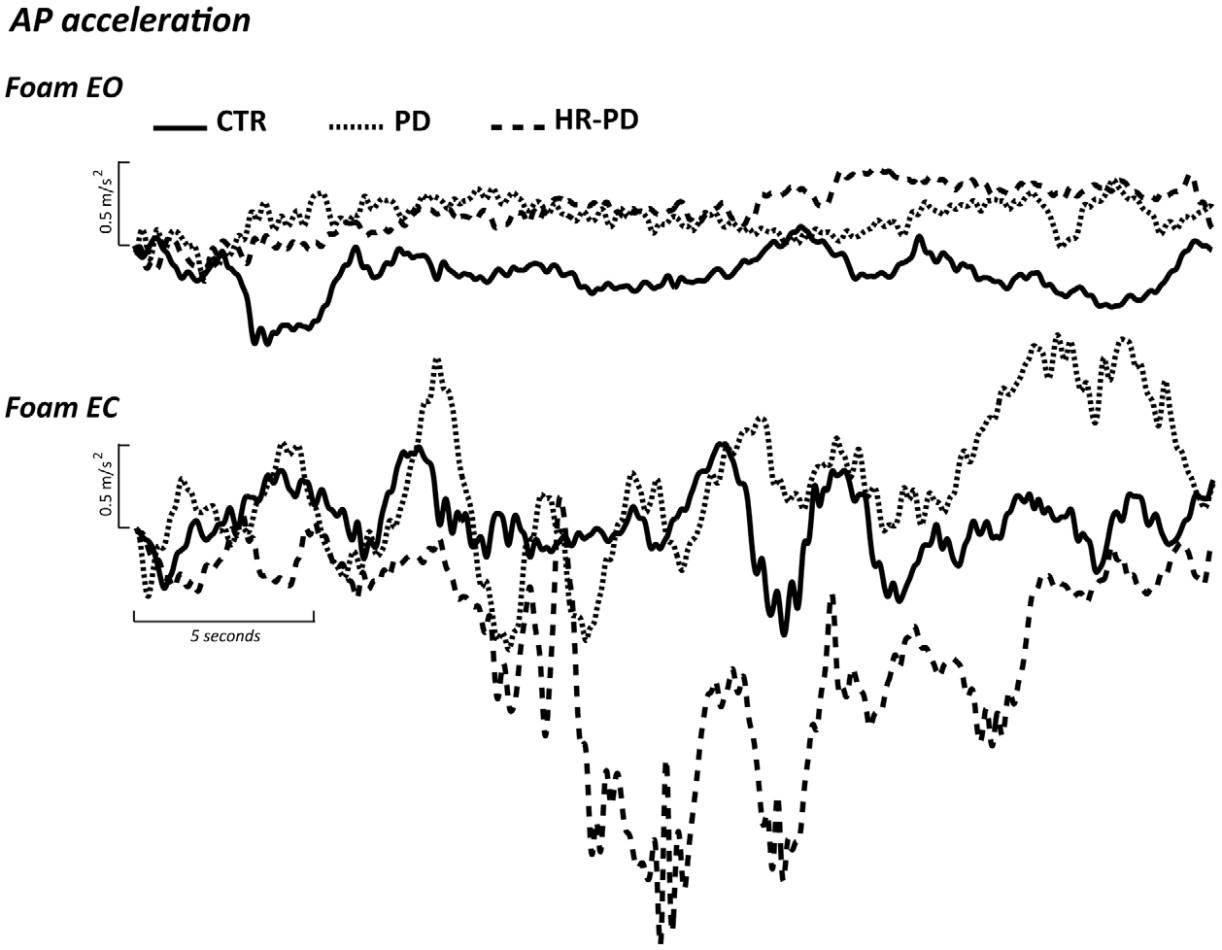
Traces of representative individuals. Traces of the anteroposterior acceleration in the eyes open (EO), top, and eyes closed (EC), bottom, foam trials for a representative subject for each group: Parkinson's disease (PD) – dotted; increased risk for PD (HR-PD) – dashed; controls (CTR) – solid line.

Representative signals are shown in figure 2 to allow a visual inspection of qualitative differences among trunk anteroposterior acceleration across groups.

In a logarithmic model the influence of demographic and clinical parameters on these two most relevant parameters was tested. RMS and JERK were both significantly influenced by cohort (as expected) and MMSE (R^2^≤46%; a logarithmic model including the ECF sway parameters presented in table 1 and figure 1 together with the MMSE lead to p-values≤0.003, thus MMSE values did not affect presented results) but not by gender, weight, length, UPDRS III, BDI, SN status and pallaesthesia score.

## Discussion

To our knowledge, this is the first report on changes in sway performance among healthy individuals with an increased risk of PD. HR-PD individuals showed an increased variability of their trunk acceleration pattern in particular under a difficult sway condition, as compared to both PD patients and controls. These results are coincident with what was recently found by Mirelman and colleagues [8] in terms of gait variability in another high risk cohort for Parkinsonism, i.e. in LRRK2 G2019S mutation carriers. Comparable to what is discussed by these authors, our findings may indicate subtle abnormalities of the central (here: balance-related) networks as manifested during challenging conditions, demonstrating decreased compensatory reserve. As proposed by Bottaro and colleagues [29], the model best explaining human body sway while quiet standing is a regular (or periodic) interplay between a fall and a stabilization phase, with an estimate of approximately 0.4 seconds per phase. Based on this model, the increased variability of the trunk acceleration pattern in HR-PD risk individuals may indicate a loss of capacity of the central balance control system which is responsible for the regularity (or periodicity) of the phases.

In addition, HR-PD individuals showed a more accentuated increase of JERK, i.e. a decrease in smoothness of sway reflecting a reduced efficiency of trunk control, with increasing sway task difficulty which also reached significance in the most challenging condition, i.e. semitandem stance on foam with closed eyes. The intermittent stabilization scheme proposed by Bottaro and colleagues [29] predicts that sway movements should be rather smooth, because they are mainly driven by intrinsic dynamics (and not by external input, or noise). In fact, an increase of JERK has repeatedly been associated with disturbance of balance in neurodegenerative disorders such as PD [5], [30], and degenerative cerebellar diseases [31]. It is intriguing to hypothesize that the HR-PD individuals with the highest values may be those who most probably will develop PD within the next years. This hypothesis will be tested during the regularly performed follow-up visits.

Interestingly, HR-PD individuals were not only different from controls regarding the abovementioned parameters but also from PD patients (all without tremor, without clinical signs of postural instability, a Hoehn&Yahr stage of ≤2.5 and a mean disease duration of approximately 4 years), who showed values comparable to the control cohort. This seems counterintuitive as PD patients definitely suffer from a postural deficit as shown by clinical and quantitative analyses [1], [10], [32], [33]. However most of the previous studies used different stance paradigms (i.e. parallel stance and not semitandem), included patients with more advanced disease course, and/or did not particularly focus on the parameters assessed here (only recently accelerometer-based trunk measures were introduced). One possible explanation for this finding could be that in the absence of relevant rigidity and bradykinesia - HR-PD individuals had UPDRS motor scores comparable to controls, see table 1 - a decrease in trunk stability has to be compensated by an increase of correction movements whereas stiffening typically associated with the OFF state of PD patients does not require such compensation. Another explanation could be as follows: Balance disturbances are particularly responsive to training effects [31], [34], [35], [36]. PD patients - who are aware of the occurrence of balance difficulties – may dispose of a daily and intensely trained balance control system which has developed some compensation strategies to postural instability. Contrary, HR-PD individuals are not aware of the ongoing disease process and thus do not specifically train their system, in particular in quite a naïve task as semitandem stance they may disclose a latent difficulty which may only occur under maximal challenge and not in everyday situations.

Whatever the reasons are for the “improvement” of variability of trunk acceleration and smoothness of sway in patients with PD compared to the HR-PD group, it seems very probable that it is not due to noise as we controlled for a number of potential confounding parameters, namely gender, weight, length, UPDRS III, MMSE, BDI, SN status and pallaesthesia score. Still, as the sway parameters included here can only reflect parts of the highly complex balance control system - which should function more properly in a prodromal PD phase than in clinical PD, and definitely deteriorates during the course of PD - changes of such parameters in the course of, e.g. a neurodegenerative process may not always follow linear curves.

### Limitations

The risk of our HR-PD individuals for developing PD is not exactly definable to date, but may be comparable to the risk of the participants included in the abovementioned LRRK2 mutation carriers study [8]. Similarly, it is most probable that only a minority of our HR-PD individuals will develop PD within the next years. Thus, the findings should be interpreted with caution as we cannot exclude that deficits observed in the HR-PD individuals might reflect, at least partly, an endophenotypic marker and not an early biomarker of PD.

### Conclusion

Balance dynamics under maximally challenging conditions - e.g. with exclusion of proprioceptive components and use of difficult stance conditions - might serve as new, sensitive biological markers of prodromal PD.
